# Anatomical Insights into Frontal Branch Preservation in Minimally Invasive Brow Lift Techniques: A Comparative Analysis of Anatomical Lift Prantl’s Suture Suspension (ALPS) Versus Gliding Brow Lift (GBL)

**DOI:** 10.1007/s00266-025-05455-x

**Published:** 2025-11-24

**Authors:** Lukas Prantl, Alexandra M. Anker, Lena Hamoshi, Vanessa Brébant, Andreas Kehrer, Silvan M. Klein, Marc Ruewe, Andreas Siegmund, Anna Wiesmeier, Jasmin Lenhard, Niklas Biermann

**Affiliations:** 1 Department of Plastic, Reconstructive and Hand Surgery, University Hospital Regensburg, Franz-Josef-Strauss-Allee 11, 93053 Regensburg, Germany; 2 IN-aesthetik, Münchener Str. 135, 85051 Ingolstadt, Germany

**Keywords:** Brow lift, Facial rejuvenation, Suspension brow lift, Anatomical Lift Prantl’s Suture Suspension (ALPS), ALPS lift, Gliding Brow Lift (GBL), Facial nerve injury

## Abstract

**Abstract:**

The brow region plays a crucial role in facial expression and appearance, making it a key aspect of facial rejuvenation procedures. This study focuses on minimally invasive brow lift techniques including the Anatomical Lift Prantl’s Suture Suspension (ALPS) and the Gliding Brow Lift (GBL). Based on a cadaveric dissection and a clinical case presentation, the study identifies the technical nuances of these procedures and highlights anatomical danger zones, with a particular focus on the frontal branch of the facial nerve. During both the ALPS and GBL procedures on fresh-frozen cadaveric specimens, sutures were placed and their relation to critical anatomical structures, such as the frontal branch of the facial nerve, was assessed. Results indicated that ALPS technique, which uses fewer sutures and employs a periosteal approach, reduces the risk of nerve entrapment and injury. In contrast, the GBL technique utilizes multiple percutaneous sutures for skin stabilization, which were observed to be in closer proximity to the frontal branch of the facial nerve. In clinical studies using the GBL technique, however, no nerve injuries have been observed so far, and if they do occur, they are only temporary with complete recovery. Nevertheless, this study highlights the importance of understanding anatomical relationships in the brow region to ensure safety and efficacy in brow lift procedures.

**Level of Evidence IV:**

This journal requires that authors assign a level of evidence to each article. For a full description of these Evidence-Based Medicine ratings, please refer to the Table of Contents or the online Instructions to Authors www.springer.com/00266.

## Introduction

Among the various aspects of facial rejuvenation, the brow region holds significant prominence due to its central role in facial expressions and overall appearance [1, 2]. The brow region is susceptible to the effects of aging which mainly manifests in sagging as a result of soft tissue laxity. These changes contribute to a tired appearance but can also impair visual fields regarding a functional perspective. Consequently, brow lift procedures have gained popularity as effective solutions for restoring a youthful brow contour while addressing functional concerns. In recent years, brow lift techniques have experienced substantial evolution, driven by advancements in surgical techniques and a deeper understanding of facial anatomy.

One novel technique is the suspension brow lift (SBL), also known as the suture or thread lift, which has gained popularity for its minimally invasive nature and rapid recovery [3]. This approach involves the strategic placement of sutures or threads to lift and reposition brow tissues, creating a rejuvenated appearance. Different dissection planes or geometric figures are described for the suture placement and anchoring method [3–5]. By utilizing specialized threads, such as barbed or non-absorbable sutures, surgeons can achieve precise elevation and fixation of the brow without the need for extensive incisions. We have refined these techniques and developed a feasible and straightforward variation using a U-shaped thread loop, which is minimally invasively inserted via cannulation. Thereby, we achieve a firm anchoring at the eyebrow, the galea aponeurotica and the periosteum. The eyebrow is anatomically repositioned using this “Anatomical Lift Prantl’s Suture Suspension (ALPS)” method described below in greater detail.

Another promising technique is the Gliding Brow Lift (GBL), which focuses on releasing and repositioning of the brow using a sliding mechanism following subcutaneous dissection [6–8]. Unlike traditional approaches involving tissue excision or permanent fixation, the GBL relies on transient transcutaneous stabilization using a hemostatic net [6]. These sutures hold the brow position and contour while preserving tissue integrity and minimize the risk of visible scarring.

Despite the growing interest in minimally invasive brow lift procedures, studies evaluating their efficacy and safety are still limited.

The aim of this article was to provide detailed step-by-step instructions on current minimally invasive brow lift techniques including ALPS and GBL by means of a cadaveric dissection and a clinical case presentation. In particular, the anatomical relation of the placed suture material to functionally relevant structures such as the frontal branch of the facial nerve was investigated.

## Materials and Methods

This anatomical study was conducted upon approval of the Ethics Committee of the University of Regensburg (23-3288-104) and according to the CONSORT (CONsolidated Standards of Reporting Trials, 2010) guidelines respecting the Declaration of Helsinki. Oral and written informed consent was obtained from the patient of the clinical case presentation.

Two hemi-faces of a fresh-frozen cadaver were surgically dissected to demonstrate two current minimally invasive brow lift techniques (ALPS and GBL). The study was conducted in collaboration with the local Institute of Anatomy, and informed consent was provided.

First, an open bicoronal approach was used to identify the frontal branch of the facial nerve on both hemi-faces to facilitate conclusions regarding the risk of its injury depending on the brow lift technique employed. A skin stapler was used for temporary skin closure.

**Fig. 1:**
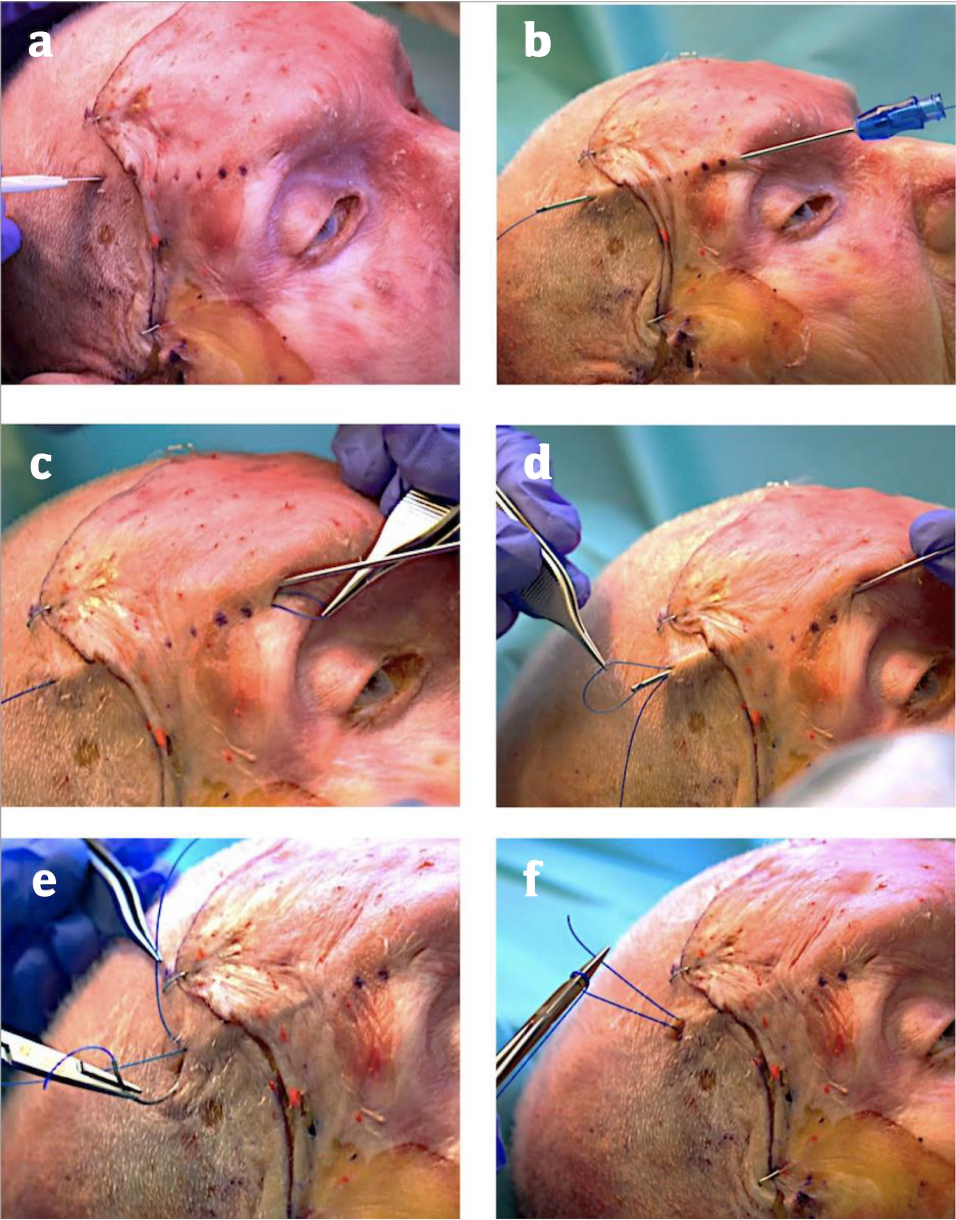
Stepwise approach of the Anatomical Lift Prantl’s Suture Suspension (ALPS). **a**: The desired vector of pull is marked by the dotted line and the blade tip points towards the incision site for suture fixation. Another stab incision is made at the superior border of the brow. **b**: A cannula is inserted retrograde from just within the brow to the fixation point posterior to the hairline in the periosteal plane. **c**: The back end of the desired suspension suture is passed antegrade through the cannula which then is pulled back to the brow region and repositioned two millimeters medially in the subcutaneous plane to create a U-shaped loop for adequate soft tissue attachment at the brow. **d**: The cannula is moved back in the periosteal plane to the exit point at the scalp and the suture is retracted. **e/f**: Fixation of the suture to the periosteum and galea. Due to its placement in the periosteal plane, the suspension suture did not come into contact with the frontal branch of the facial nerve. Nota bene: The coronal approach illustrated here is made solely for demonstration purposes to highlight the distance of the suspension suture to the frontal branch of the facial nerve

Then the ALPS was performed followed by the GBL technique (Figs. 1 and 5). To track the sutures’ traces through the tissue, red-colored nail polish on the needles tip was used.

**Fig. 2:**
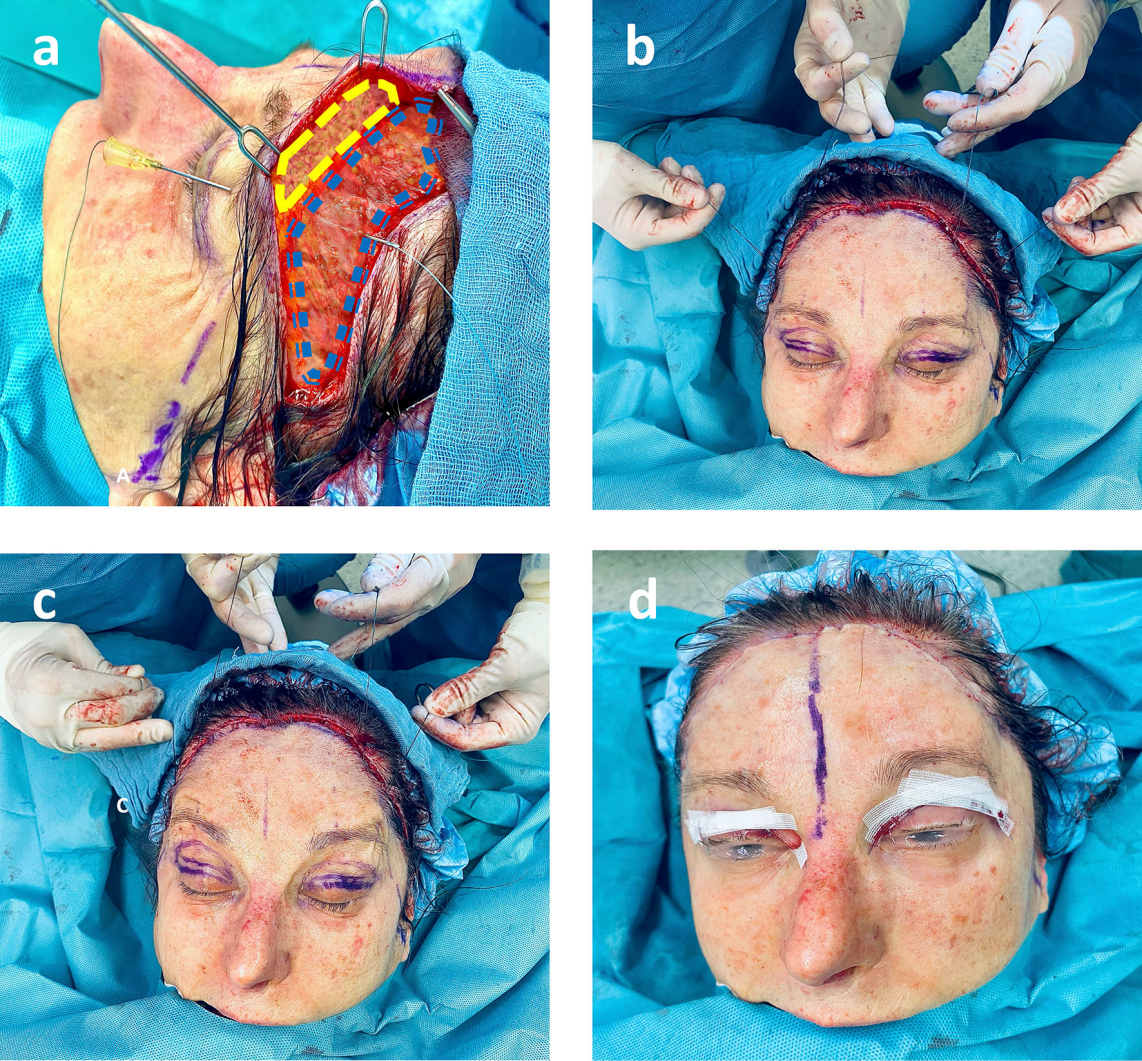
Clinical case presentation of the Anatomical Lift Prantl’s Suture Suspension (ALPS) in combination with a subcutaneous open forehead lift and blepharoplasty. In this case, the ALPS technique was demonstrated during a subcutaneous open forehead lift to enhance visualization of the dissection planes for educational purposes. It is important to note that the ALPS is typically performed as a minimally invasive brow lift technique without requiring a bicoronal approach. Intraoperative photographs of **a**: placement of a 20-G (70 mm) cannula (Sterican, B. Braun, Germany) with suspension suture (FiberWire® 3-0, Arthrex, Florida, USA) in the periosteal plane. The blue dotted line indicates the frontalis muscle, and the yellow dashed line marks the forehead flap elevated in the subcutaneous plane. **b/c**: In total, four suspension sutures are placed within the brows bilaterally and the vector of pull is demonstrated. **d**: Clinical result after wound closure with a significantly elevated brow position. The dark-colored marking corresponds to the midline

Supplementary intraoperative photographic material of a clinical case demonstrating the ALPS is presented (Fig. 2).

## Results

### Surgical Technique

#### Anatomical Lift Prantl’s Suture Suspension (ALPS) (Figs. 1, 2, 3 and 4)

**Fig. 3:**
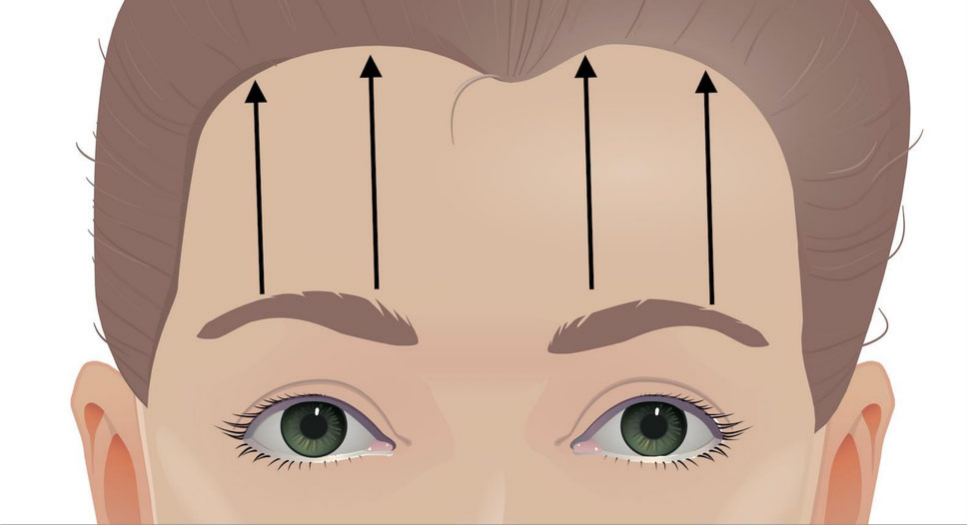
Schematic illustration of the Anatomical Lift Prantl’s Suture Suspension (ALPS). Typically one or two suspension sutures are placed on each side of the forehead. The black arrows demonstrate the course of the suspension suture beneath the skin. One suture is placed on each side of the forehead in the medial third of the brow, while the other one is placed at the apex of the brow which is located at the level of the crista temporalis

**Fig. 4:**
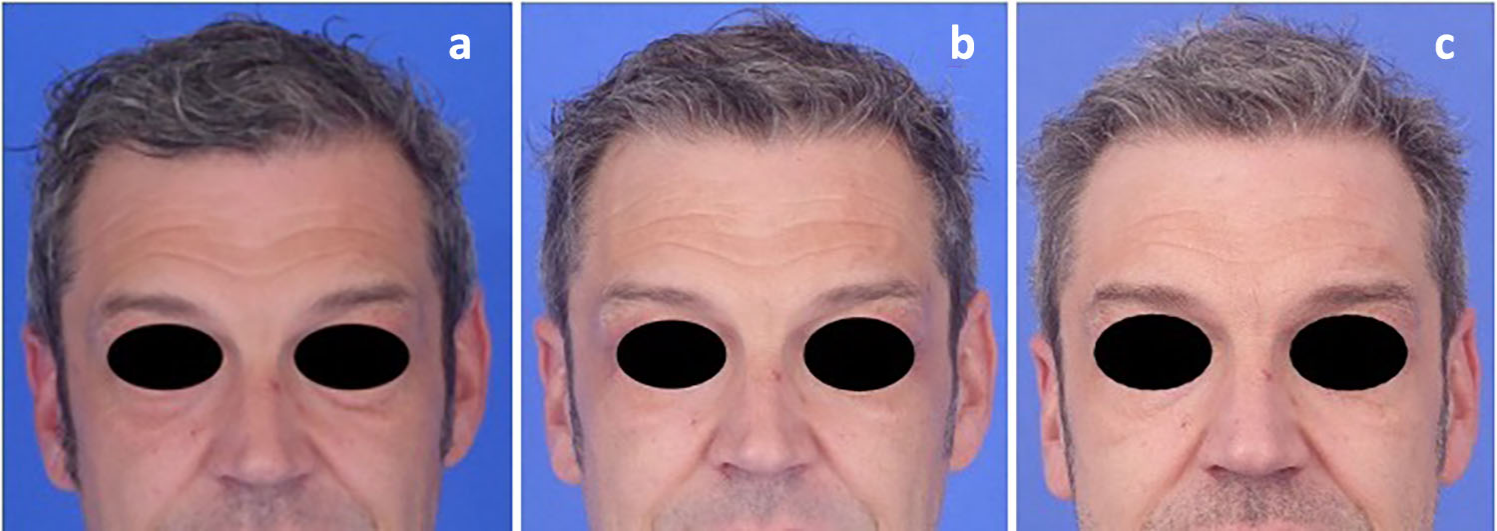
Medium- and long-term case study following the ALPS technique. **a** Preoperative view. **b** Six-week postoperative view. **c** Ten-month postoperative view. The photographs demonstrate a successfully elevated brow position with stable medium- and long-term results. To objectify the outcome, a ratio was calculated (hairline to cranial border of the eyebrow ÷ cranial border of the eyebrow to nasal root). In cases of sufficient brow elevation, this ratio should decrease after the procedure. Six measurements were taken at each time point to calculate a mean ratio: preoperatively **a** 0.75, at 6 weeks **b** 0.74, and at 10 months **c** 0.69, indicating objective eyebrow elevation with sustained stability over time

The ALPS method provides a precise and standardized anatomical approach. What distinguishes ALPS is its strict focus on specific anatomical planes, especially the periosteal layer, making it unique compared to other suspension techniques that often fail to clearly define the suture insertion plane.

ALPS may be performed under local anesthetic infiltration or general anesthesia in a beach-chair position. The desired vector of pull is marked on the skin in accordance with the patient’s desire and functional concern in the upright position. Typically, one or two suspension sutures are placed on each side of the forehead in a single procedure (Fig. 3).

To begin the procedure, mark the midline as a reference line for orientation. Then, mark the exit point of the supratrochlear and supraorbital nerves, approximately 15 and 20 mm lateral to the midline, respectively. This ensures precision in avoiding sensory nerve interference. Next, mark the crista temporalis to define the lateral boundary. These markings serve as essential landmarks to guide the accurate placement of incisions and sutures during the procedure.

For each suture, a one-centimeter incision is made slightly posterior to the hairline down to the level of the periosteum and a stab incision is placed down to the subcutaneous plane close to the superior border of the lateral brow. Guidance of the permanent suture (FiberWire® 3-0, semicircular periosteal needle, Arthrex, Florida, USA) through the tissue is established using a 20-G cannula with a length of 70 mm (Sterican, B. Braun, Germany) inserted from the brow to the hairline in the deep periosteal plane. The back end of the suture is passed antegrade through the cannula. With the suture secured cranially, the cannula is pulled back and repositioned within the brow taking a 2-mm bite in the subcutaneous plane and then returned to the scalp incision in the periosteal plane creating a small U-shaped loop. Feel the needle tip with your index finger as you advance to ensure it remains in the correct layer. Retract the suture first and then remove the cannula. For suture fixation, the galeal plane and periosteum are chosen at the hairline incision as the counter bearing tissues to hold the brows weight and ensure long-term stability (Figs. 1 and 2).

Figure 4 displays a medium- and long-term case study of the ALPS technique, showing stable brow elevation over ten months.

#### Gliding Brow Lift (GBL) (Fig. 5)

**Fig. 5:**
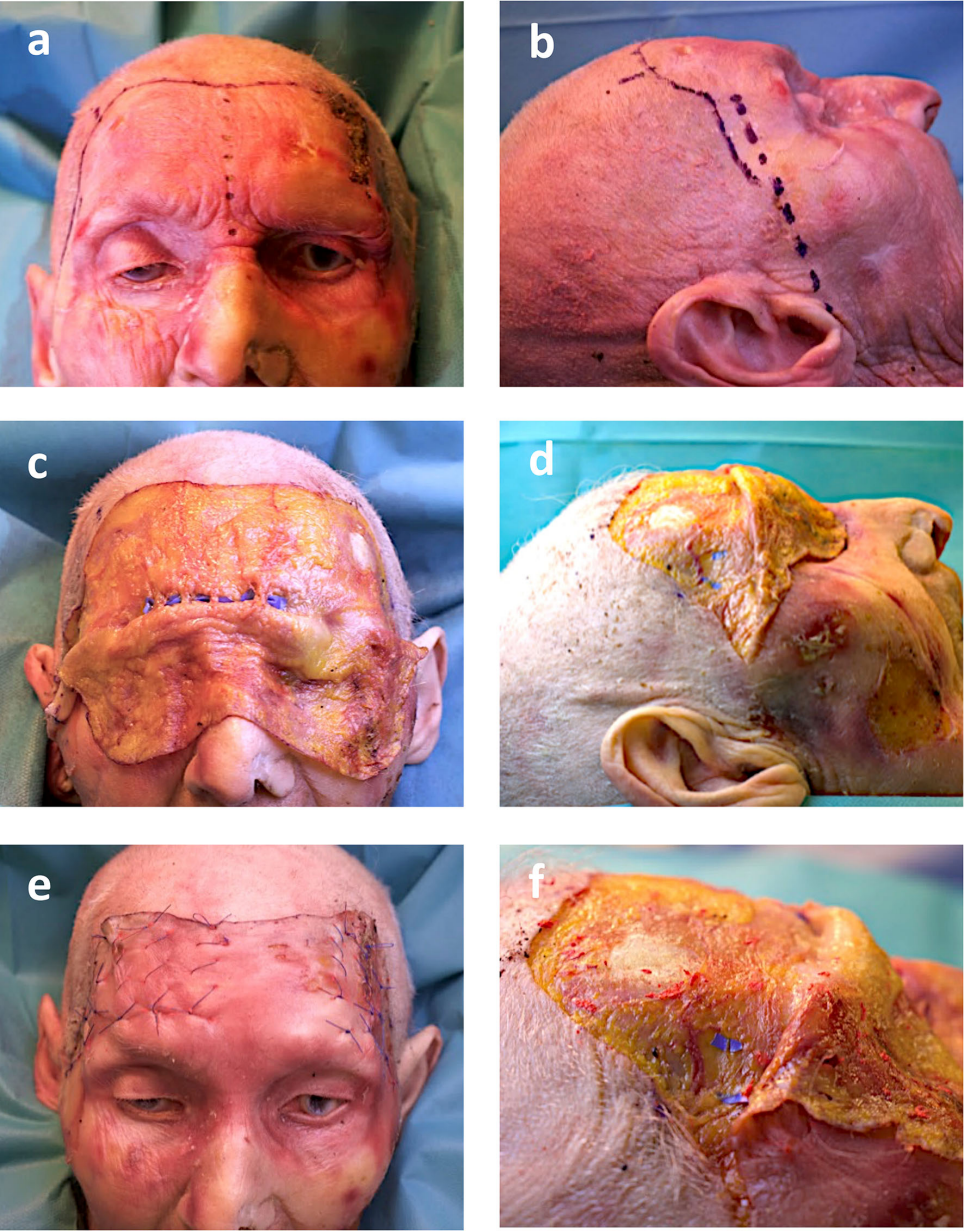
Sequential approach of Gliding Brow Lift (GBL) and anatomical dissection. **a**/**b**: In order to demonstrate the anatomical relation of the hemostatic net sutures to underlying functionally relevant structures, an open forehead lift is planned first using a bicoronal approach. The dark-colored dotted line represents the anatomical midline, while the dark-colored dashed line corresponds to Pitanguy’s line, indicating the expected course of the frontal branch of the facial nerve. **c**: Subcutaneous dissection is followed by marking the bilateral supraorbital and supratrochlear nerves (blue rubber). **d**: Similarly, the frontal branch of the facial nerve is identified and marked (blue rubber). **e**: The skin envelope is temporarily retracted to perform the GBL. Inked hemostatic net sutures are applied. **f**: After suture removal, red dots indicating their previous location are visualized in close proximity to the frontal nerve branch (blue rubber)

In our clinic, GBL procedure is performed under general anesthesia and in beach-chair position. Preoperative markings include the area desired to lift. A minimum of two stab incisions per side are placed within or slightly posterior the hairline. Hydrodissection using epinephrine containing tumescent solution is performed within the subcutaneous plane. Blunt dissection in vertical and horizontal direction is achieved extending just below the eyebrow over the orbital rim. The entire frontal area is widely dissected laterally expanding toward the zygomatic arch. Straight and L-shaped dissectors out of the arthroscopic armamentarium can be used to ensure blunt preparation. The desired lifted position of the brow and the frontal skin flap is adjusted using Gillies hooks and fixed using a 4-0 horizontal nylon suture (Ethilon 4-0, Ethicon, Germany). The hemostatic net is applied vertically in several rows to obliterate dead space and prevent hematoma formation (Ethilon 4-0, Ethicon, Germany) (Fig. 5).

## Discussion

The aim of this article was to provide detailed step-by-step instructions on ALPS and GBL representing two promising minimally invasive brow lift techniques using a human head specimen and a clinical case presentation. Particular attention was paid to the anatomical relation of the dissection planes of the employed techniques to the frontal branch of the facial nerve.

Our literature review indicated significant differences among various traditional and modern brow lift techniques in terms of invasiveness, efficacy, risk of wound healing complications, extent of scarring and alopecia, potential for hairline alteration, and the risk of injury to the frontal branch of the facial nerve [9–12]. With up to 2 % incidence, the latter is a rare but significant complication, as paralysis of the frontalis muscle results in brow ptosis, which directly contradicts the desired outcome of the brow lift procedure [13].

Our results have shown that during the GBL, the percutaneous placement of multiple sutures that secure the skin to the frontalis muscle penetrating all layers, may result in a close anatomical relationship with the frontal branch of the facial nerve. This might raise concerns about the potential risk of motor nerve injury. However, it is important to note that in the original description of the GBL technique by Viterbo et al., no instances of permanent paralysis or asymmetrical movement were reported in 124 performed operations [6].

Despite the close proximity of the sutures to the frontal branch of the facial nerve observed in our cadaver study, the suture material used (5-0 and 4-0) does not seem to cause permanent nerve lesions in clinical practice. Additionally, the sutures in Viterbo et al.'s study were removed after 48 hours [6]. It is unknown what level of nerve damage might result from this temporary compression. In Sunderland class I and II injuries, the epineurium, which surrounds the myelin sheath of the axon, remains intact. With this membrane preserved, the injured axons are able to regenerate and reconnect with their original motor endplates, allowing for complete recovery of nerve motor function [14]. Evidence from an animal study involving peroneal nerve transfixion through a suture stitch in six rats demonstrated normalization of walking functionality after suture removal within 48 hours and complete recovery within 13 days [15]. Consequently, it is possible that subtle, temporary nerve compression injuries were not reported due to their transient nature or because they eventually resolved without significant clinical consequences.

Given that nerve injury can result in permanent brow ptosis, which is contrary to the patient's desired surgical outcome, it is imperative that each surgeon considers individually whether to avoid suturing in the danger zone of the frontal branch during any brow lift technique. While this study has its focus on the risk of frontal branch injury, it is also important to consider that myoparalysis might result from direct damage to the frontal muscle caused by dissection errors or tissue pressure from suture entrapment.

In contrast to the GBL and other minimally invasive brow lift techniques, the ALPS involves placement of usually one or two suspension sutures only on each side of the forehead.

Since fewer sutures in the periosteal plane are placed in ALPS technique, the inherent risk of nerve injury is low. While the preparation planes vary among the described SBL techniques in the literature, the ALPS method relies on a deep placement of the suture in the periosteal plane to minimize the risk of interference with the frontal branch. Most authors describe the frontal branch of the facial nerve to travel within loose areolar tissue under the superficial temporal fascia or within the temporoparietal fascia [16–18]. In concordance with other anatomical zones of adherence located throughout the body, the superficial temporal fascia adheres to the deep temporal fascia at the level of the temporal crest. This anatomical complexity presents a challenge, particularly to the inexperienced surgeon, as it renders the identification and maintenance of a correct and safe dissection plane even more difficult. The periosteal approach minimizes the risk of injury to the frontal branch of the facial nerve as it is easy to identify, easy to dissect, and nearly bloodless. Furthermore, compared to other brow suture suspension techniques, another advantage of the ALPS technique with a placement of the suture in the periosteal layer is that the suture remains invisible. In contrast, when the suture is placed more superficially in the subcutaneous plane, it can sometimes become noticeable in patients with a thin skin envelope.

Regarding the optimal suture material for the ALPS procedure, our experience highlights several key advantages of FiberWire® 3-0 (Arthrex, Florida, USA). Its semicircular periosteal diamond point needle allows for precise insertion, while the suture’s combination of high strength and soft texture minimizes tissue irritation, making it an excellent choice for suture suspension brow lifts. The suture itself is highly durable. Based on the high tensile strengths demonstrated in flexor tendon repairs using FiberWire® sutures, we expect this material to be the most appropriate [19]. The secure anchoring provided by the U-shaped loop at the brow and the placement of the knot at the galea and periosteum ensures stability. This is what sets the ALPS technique apart from other thread lift sutures [20]. The ALPS method offers several distinct advantages that make it a highly promising and accessible surgical technique. It is simple to perform, ensuring that surgeons can quickly learn and apply it with confidence. As a minimally invasive procedure, the ALPS method is associated with a short recovery time, allowing patients to promptly return to their daily activities. Additionally, it leaves inconspicuous scars, which enhances patient satisfaction with the cosmetic results. The ALPS method offers a precise and standardized anatomical approach. A key distinction of ALPS is its adherence to specific anatomical planes, particularly the periosteal layer, which sets it apart from other suspension techniques that often lack clear definition of the suture insertion plane. By providing a well-defined and reproducible technique, ALPS ensures consistency and facilitates its implementation by other colleagues. It allows for easy corrections or adjustments if necessary. Another advantage of the ALPS technique is that it represents a truly minimally invasive procedure with low patient downtime. In contrast, the GBL is often described as minimally invasive due to its small skin incisions, but in reality involves an extensive dissection comparable to the endoscopic brow lift. This discrepancy between minimal scars and wide surgical dissection should be taken into account, particularly in the context of patient counseling.

At this stage, however, we cannot ascertain that the technique provides comparable stability to other more invasive procedures involving extensive dissection and dissolution of retaining ligaments. The ALPS technique and suspension brow lifts in general are best suited for patients with moderate skin thickness, mild to moderate brow ptosis. For severe ptosis with significant skin excess, more invasive techniques may offer greater efficacy but come with increased swelling, longer downtime, and higher complication rates, underscoring the importance of appropriate patient selection.

The combination of simplicity, effectiveness, and flexibility, however, makes the ALPS method a strong choice for eyebrow repositioning, but its long-term clinical efficacy remains to be proven.

As a future perspective, further studies are needed to establish the long-term outcomes and stability of the ALPS technique. This manuscript provides a step-by-step guide along with insights into the technical pearls and safety of the method, laying the groundwork for future research and clinical evaluation. Ultimately, not every technique works equally well in every surgeon's hands, and both the experience and preference of the surgeon, as well as patient-specific factors, should be taken into consideration. Therefore, a tailored approach that accounts for both the surgeon's expertise and the unique characteristics of the patient is essential for optimal outcomes.

## Conclusion

Compared to traditional open brow lift techniques, novel approaches for addressing brow ptosis, such as the GBL or the ALPS method, are significantly less invasive. However, minimally invasive procedures can present the challenge of a limited visual field. This cadaveric dissection study demonstrated that sutures placed during minimally invasive brow lift techniques might interfere with the frontal branch of the facial nerve. Current clinical data, however, report functionally relevant brow ptosis due to frontal branch injury or direct myoparalysis as a rare complication. To improve patient outcomes and enhance safety, the authors recommend deep dissection planes at the layer of the periosteum, as used in the ALPS method, to minimize nerve interference. Additionally, avoiding established "danger zones" where the frontal branch of the facial nerve is most vulnerable further reduces complications.
